# Editorial: Reviews and advances on the role of membrane trafficking in cancer

**DOI:** 10.3389/fcell.2025.1734267

**Published:** 2025-11-14

**Authors:** Christophe Lamaze, Stéphane Gasman, Ewan MacDonald

**Affiliations:** 1 Institut Curie - Centre de Recherche, PSL Research University, Membrane Mechanics and Dynamics of Intracellular Signalling Laboratory, Paris, France; 2 Institut National de la Santé et de la Recherche Médicale (INSERM), U1339, Paris, France; 3 Centre National de la Recherche Scientifique (CNRS) UMR3666, Paris, France; 4 Centre National de la Recherche Scientifique, Université de Strasbourg, Institut des Neurosciences Cellulaires et Intégratives, Strasbourg, France; 5 Institut de Recherche en Infectiologie de Montpellier (IRIM), Université de Montpellier CNRS UMR 9004, Montpellier, France

**Keywords:** endocytosis, exocytosis, extracelluar vesicles, membrane trafficking, cancer

## 1 Introduction

Membrane trafficking refers to the regulated transport of biomolecules between distinct cellular compartments within membrane-enclosed structures. This fundamental process not only ensures essential cellular functions, such as nutrient uptake and secretion of signalling factors like hormones, but also regulates signalling through the compartmentalization of receptors and effectors. Broadly, membrane trafficking can be divided into endocytic events, which internalize material from the extracellular space, and exocytic events, which deliver vesicles toward the cell surface (Yarwood et al., 2020).

Interest in understanding the organization of membrane trafficking pathways dates back to the mid-20th century (Palade, 1975). For their pioneering work elucidating the architecture and organisation of this system, George Palade, Christian de Duve, and Albert Claude were awarded the 1974 Nobel Prize in Physiology or Medicine. The field gained renewed recognition in 2013, when James Rothman, Randy Schekman, and Thomas Südhof were honoured with the Nobel Prize for their discoveries of the machinery regulating vesicular trafficking. Through elegant biochemical and genetic studies, they uncovered the fundamental principles that define this now mature field (Novick and Schekman, 1979; Fries and Rothman, 1980; Novick et al., 1980). Yet, membrane trafficking continues to yield new surprises. Its ever growing molecular complexity and regulatory networks attest to this.

Given its central role in cellular homeostasis, it is not surprising that mutations or dysregulations in trafficking components underlie a wide range of diseases, including Parkinson’s disease and haemophilia (Yarwood et al., 2020). In the context of cancer, membrane trafficking is often subverted to enhance cellular fitness and adaptability (Sigismund and Scita, 2018). This Research Topic in *Frontiers in Developmental Cell Biology* highlights ongoing efforts to elucidate the complex and evolving roles of membrane trafficking in cancer.

Although dysregulated membrane trafficking is not yet considered one of the canonical hallmarks of cancer (Hanahan, 2022), growing evidence indicates that the trafficking system is extensively reprogrammed to facilitate disease progression. Broadly, this can be categorized into three major themes.

## 2 Metabolic reprogramming via endocytic pathways

Enhance endocytic activity helps cancer cells meet their elevated metabolic demands. Internalized material is processed within lysosomes, where it is degraded and recycled into the building blocks of proteins, lipids, and metabolites required for growth (Commisso et al., 2013). Lysosomes have thus emerged as central organelles in metabolic regulation (Settembre and Perera, 2024). A parallel phenomenon is observed in tumour-infiltrating macrophages, which engulf apoptotic cells and debris. In this Research Topic, Yan et al. discuss how this influx of material drives macrophage metabolic reprogramming, reshaping immune responses and influencing the tumour microenvironment.

## 3 Dysregulated secretion and vesicular communication

Increased secretion of hormones, cytokines, and extracellular vesicles (EVs) promotes metastatic progression by remodelling the pre-metastatic niche and modulating intercellular signalling, including immune interactions (Madden et al., 2020; Kalluri and McAndrews, 2023).

In this Research Topic, three contributions explore this dimension. Hu et al. describe a novel form of vesicular communication mediated by *cytonemes*, specialized filopodial protrusions that establish direct contacts with neighbouring cells, enabling long-distance transfer of bioactive molecules. Kailasam Mani et al. review the formation and function of tumour-derived extracellular vesicles (TEVs) and their roles in intercellular communication. In particular, they highlight their recent findings that physical stress can induce EV formation in a caveolin-1-dependent manner (Saquel et al., 2024). Finally, Streit et al. examine the molecular mechanisms underlying the dysfunction of the regulated secretory pathway in neuroendocrine tumours, which contributes to clinical complications.

## 4 Altered surface composition and signalling dynamics

A third major theme centres on how dysfunctional membrane trafficking reshapes the plasma membrane landscape, altering lipids and receptor composition to promote cancer progression (MacDonald et al., 2025). Guo et al. report that the ESCRT subunit CHMP7, a regulator of lysosomal degradation, has predictive value for patient prognosis and may play a role in anti-tumour immunity. Patat et al. propose that a deeper understanding of the higher-order organization and collective behaviour of trafficking regulators at the organelle level will be essential to grasp how trafficking plasticity supports tumour adaptability.

Through this Research Topic, we aim to highlight some emerging insights into how membrane trafficking contributes to cancer development and progression. A more comprehensive understanding of these mechanisms will be key to identifying new therapeutic vulnerabilities and developing strategies to overcome drug resistance.
